# Supplementary material for: Comparative genomics provides new insights into the diversity, physiology, and sexuality of the only industrially exploited tremellomycete: Phaffia rhodozyma
Source: BMC Genomics. 2016 Nov 9;17:901. doi: 10.1186/s12864-016-3244-7 (PMC5103461; doi:10.1186/s12864-016-3244-7)
Supplement: Additional file 6: — List of orphan genes with links to PFAM (related to Additional file 1: Table S1). (ZIP 1428 kb) [file 12864_2016_3244_MOESM6_ESM.zip › PFAM_UNIQUE/G04325_P.html]

#                                                                            --- full sequence --- -------------- this domain -------------   hmm coord   ali coord   env coord
  
# target name        accession   tlen query name           accession   qlen   E-value  score  bias   #  of  c-Evalue  i-Evalue  score  bias  from    to  from    to  from    to  acc description of target
  
#------------------- ---------- ----- -------------------- ---------- ----- --------- ------ ----- --- --- --------- --------- ------ ----- ----- ----- ----- ----- ----- ----- ---- ---------------------
  
G04325\_P             -            427 PNRC                 PF15365.1     58    0.0019   16.8   2.2   1   2       2.7     4e+03   -3.4   0.2     4    11   339   346   325   351 0.68 - Proline-rich nuclear receptor coactivator  
G04325\_P             -            427 PNRC                 PF15365.1     58    0.0019   16.8   2.2   2   2   4.8e-06    0.0071   15.0   0.2     5    24   403   422   400   424 0.93 - Proline-rich nuclear receptor coactivator
